# The synthetic inhibitor of Fibroblast Growth Factor Receptor PD166866 controls negatively the growth of tumor cells in culture

**DOI:** 10.1186/1756-9966-28-151

**Published:** 2009-12-11

**Authors:** Gianfranco Risuleo, Marina Ciacciarelli, Mauro Castelli, Gaspare Galati

**Affiliations:** 1 Dipartimento di Genetica e Biologia Molecolare, Sapienza Università di Roma, Italy; 2 Regina Elena Cancer Institute, Roma, Italy; 3 Dipartimento di Chirurgia "Pietro Valdoni", Roma, Italy

## Abstract

**Background:**

Many experimental data evidence that over-expression of various growth factors cause disorders in cell proliferation. The role of the Fibroblast Growth Factors (FGF) in growth control is indisputable: in particular, FGF1 and its tyrosine kinase receptor (FGFR1) act through a very complex network of mechanisms and pathways. In this work we have evaluated the antiproliferative activity effect of PD166866, a synthetic molecule inhibiting the tyrosin kinase action of FGFR1.

**Methods:**

Cells were routinely grown in Dulbecco Modified Eagle's medium supplemented with newborn serum and a penicillin-streptomycin mixture.

Cell viability was evaluated by Mosmann assay and by trypan blue staining. DNA damage was assessed by *in situ *fluorescent staining with Terminal Deoxynucleotidyl Transferase dUTP nick end labeling (TUNEL assay).

Assessment of oxidative stress at membrane level was measured by quantitative analysis of the intra-cellular formation of malonyl-dialdheyde (MDA) deriving from the decomposition of poly-unsaturated fatty acids.

The expression of Poly-ADP-Ribose-Polymerase (PARP), consequent to DNA fragmentation, was evidenced by immuno-histochemistry utilizing an antibody directed against an N-terminal fragment of the enzyme.

**Results:**

The bioactivity of the drug was investigated on Hela cells. Cytoxicity was assessed by the Mosmann assay and by vital staining with trypan blue. The target of the molecule is most likely the cell membrane as shown by the significant increase of the intracellular concentration of malonyl-dihaldheyde. The increase of this compound, as a consequence of the treatment with PD166866, is suggestive of membrane lipoperoxidation. The TUNEL assay gave a qualitative, though clear, indication of DNA damage. Furthermore we demonstrate intracellular accumulation of poly-ADP-ribose polymerase I. This enzyme is a sensor of nicks on the DNA strands and this supports the idea that treatment with the drug induces cell death.

**Conclusions:**

Data presented in this work show that PD166866 has clear antiproliferative effects. The negative control of cell proliferation may be exerted through the activation of the apoptotic pathway. The results of experiments addressing this specific point, such as: evaluation of DNA damage, lipoperoxidation of the cell membrane and increase of expression of PARP, an enzyme directly involved in DNA repair. Results suggest that cells exposed to PD16866 undergo apoptosis. However, concomitant modes of cell death cannot be ruled out. The possible use of this drug for therapeutic purposes is discussed.

## Background

The dys-regulation of growth factor expression leads to alterations of cell functions such as growth control and proliferation [1,2]; as a matter of fact the role of these factors as well as that of their tyrosine kinase receptors in growth regulation is now a well established notion. This action is exerted through a myriad of mechanisms and pathways and their involvement in biological processes ranging from differentiation to apoptosis has been amply demonstrated [3-6].

The aim of this work was to evaluate the effect of a synthetic molecule, PD166866, which is an inhibitor of the tyrosine kinase function exerted by FGFR1. In addition to PD166866 other tyrosine kinase inhibitor molecules, such as SU 4984 and SU 5402 have been described. These compounds show a very high selectivity towards FGFR1 and inhibit the auto-phosphorylation activity of FGRF1, however PD166866 shows an about 100-fold higher activity [7]. Other biological activities have been ascribed to these compounds and it is generally accepted that they may find a possible application for the control of proliferation both of normal and tumor cells [8-10]. The results presented here extend a previous study where the activity of PD166866 was assayed on a normal murine fibroblast cell line in culture [10]. The impact of this drug on the overall cell metabolism was also investigated in a previous work from our laboratory [11].

Here we evaluate the bioactivity of the drug versus a human tumor cell line (HeLa). The growth inhibition monitored in this study strongly suggests that it may derive from DNA damage and activation of cell death processes most likely of apoptotic nature. Therefore a future clinical use for the control of proliferative pathologies may be envisaged.

## Methods

### Growth and maintenance of HeLa cells

Cells were maintained in DMEM (Dulbecco's Modified Eagle's Medium - high glucose), supplemented with newborn bovine serum [final concentration (f.c.) 10%], penicillin-streptomycin (10000 U/ml) and glutamine (2 mM); the pH of the medium was 7.2 and incubation was at 37°C in a 5% CO_2 _atmosphere. Cells were routinely passaged when confluent.

### Assessment of cell viability and lipoperoxidation assay

Cell viability was evaluated by the colorimetric Mosmann assay [12] which is a quantitative method measuring the level of mitochondrial damage. The MTT [3-(4,5-dimetiltiazol-2-yl)-2,5-difenil tetrazolium-bromide] is a yellow water soluble salt which is converted into insoluble purple salts formed by the active dehydrogenases present in the mitochondria of vital cells. Absorbance values measured at 570 nm provide the number of vital cells. The cell survival data were validated by vital staining with trypan blue performed by a standard laboratory protocol.

A commercial kit (LPO-586; Oxis Health Research Products Portland, Or. USA) was used to assess the oxidative stress at membrane level. Briefly, the assay is based on a quantitative analysis of the intra-cellular formation of malonyl-dialdheyde (MDA) which derives from the decomposition of poly-unsaturated fatty acids. The MDA molecule reacts with a chromogenic compound (N-methyl-2-phenylindole) thus forming a stable chromophore. Absorbance at 586 nm is directly transformed in intracellular concentration of MDA [13].

### TUNEL assay and analysis of the DNA fragmentation

The activation of the endogenous DNases is one of the consequences of cell death causing the formation of single strand nicks and eventually fragmentation of DNA. The DNA ruptures may be evidenced by *in situ *labelling. Cell nuclei are permeabilized, fluorescent dUTP is added and terminal-deoxynucleotide-transferase conjugates the nucleotide where the sugar-phosphate backbone is interrupted. Fluorescence intensity provides a qualitative idea of DNA damage [14].

### Immunolocalization of Poly-ADP-Ribose-Polymerase (PARP)

The enzyme PARP is activated in response to DNA fragmentation. The immunolocalization of PARP was performed as previously published [15]. Briefly, HeLa cells were treated with PD166866 for 24 hours, the growth medium was removed, the cells were washed with PBS and fixed for 1 hour at 25°C adding a freshly made paraformaldheyde solution (4% in PBS). Samples were washed again with PBS and the endogenous oxidases were blocked for 2 minutes in the dark. Further washes with PBS followed and blocking the unspecific sites was done for 1 hour at 25°C. PARP was evidenced by immunolocalization utilizing a polyclonal antibody (PARP H-250 Santa Cruz Biotechnology, Inc.), directed against the N-terminal proteolytic fragment. Immuno-reaction was revealed by a secondary anti-rabbit antibody after incubation for 16 hours at 4°C. After exhaustive washing with PBS the samples were incubated for 30 minutes in solution ABC (Vectastain ABC-POD Elite, PK-6101 kit, used according the supplier's recommendations). Eventually, DAB (3,3'-Diaminobenzidine) was added and the samples were incubated for 10 minutes in the dark. The samples were washed again the plates were sealed and ready for microscopic observation (Zeiss Axiophot).

### Statistical analysis

Data were obtained by at least three independent experiments. Statistical analysis was done by one way analysis of variance (ANOVA) followed by a comparative LSD test (Least Significant Difference). Results were considered significant when p < 0.05.

## Results

### Cytotoxicity of PD166866 on HeLa cells in culture

**Figure 1:**
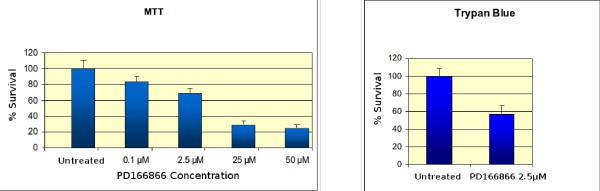
**Assessment of cell survival after treatment with PD166866**. Cells were treated with PD166866 for 24 hours at the indicated concentrations. At the end of the treatment, the samples were subjected to the Mossman assay (right panel). Alternatively after treatment cells were stained with trypan blue according to standard laboratory procedures (left panel). In this latter case only the survival at 2.5 μM is reported.

We explored the dose/response effect of HeLa cells exposed to a relatively broad range of PD166866 concentrations (0.1 - 50 μM). Cells were treated for 24 hours with the drug and their vitality assessed by the MTT assay [12]. A significant reduction of vital cells can be monitored already at 2.5 μM concentration (Figure 1, left panel). The loss of viability seems to stabilize at 25 μM (about 25% survival) with no further decrease at a 50 μM concentration of drug. This result may indicate the presence of a cell subpopulation, intrinsically resistant to the drug. This result was confirmed by vital cell count with trypan blue (only the data obtained at 2.5 μM of drug is shown; Figure 1, right panel). The negative effect of PD166866 on the cell growth was already observed in a previous works performed on 3T6 cells: a stabilized murine fibroblast line [10,11]. The results presented here validate those already published and, as far as cell survival is concerning, no difference can be monitored on HeLa in comparison to 3T6 cells in matching experiments also run in this work (not shown). Interestingly, as observed in a former study, HeLa cells showed a significantly higher sensitivity than murine cells towards resveratrol, a natural product showing both cytotoxic and antiviral properties [16]. One way to rationalize this data is that the cellular/molecular target of the two drugs could be different.

The Mosmann assay [12] indicates membrane damage, essentially at mitochondrion level. Therefore, we investigated the possibility that PD166866 may be detrimental to the membrane integrity by lipoperoxidation assays [13].

### Lipoperoxidation shows that PD166866 causes membrane damage

The lipoperoxidation assay is a very powerful tool to evaluate in a quantitative manner the membrane damage deriving from phenomena of oxidative stress. The formation of poly-unsaturated acids, consequent to this stress, causes the formation malonyl-dihaldeyde (MDA) and of 4-hydroxyhalkenals. The concentration of intracellular MDA, a compound normally not found in the cytoplasm, is correlated directly to the extent of the membrane damage [13]. In this experiment we treated the cells with PD166866 (50 μM) or with H_2_O_2 _which, like other oxygen reactive species is a very well known inducer of oxidative stress in animal and plant cell as well as in diverse pathologies [17-19].

**Figure 2:**
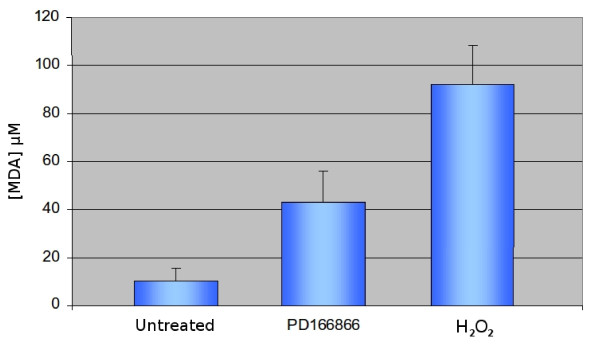
**Intracellular concentration of malonyl-dihaldehyde (MDA) after treatment with PD166866**. Cells were treated with the drug (50 μM) for 24 hours and processed for the membrane lipoperoxidation test. The intracellular concentration of MDA is over 4-fold higher in cells treated with the drug (central bar) as compared to untreated control cells (left bar). This indicates membrane damage due to oxidative stress.

The results of Figure 2 (central bar) show that that treatment with the drug causes an over 4-fold increase of the intracellular concentration of MDA: thus PD166866 induces an oxidative stress with consequent membrane damage. However, one should not be misled by the much higher level of MDA generated by H_2_O_2 _(Figure 2 left bar) since the concentration and the power of this compound is by no means comparable with that of PD166866 in this experimental context. Finally, it is known that an uncontrolled oxidative stress may lead to apoptotic cell death [20,21]. Therefore, we analyzed an additional marker diagnostic of apoptosis: DNA damage.

### DNA damage and cell death assessed by fluorescent TUNEL staining

The TUNEL assay is an experimental protocol allowing the detection of DNA fragmentation. The specificity of this assay has been disputed but modifications done to the original method [21] improved its accuracy [22]. Therefore, it is generally accepted that the correct execution of the TUNEL protocol mainly labels DNA fragmentation in very advanced phases of apoptosis [23,24] thus evidencing cells that have sustained severe DNA damage.

**Figure 3:**
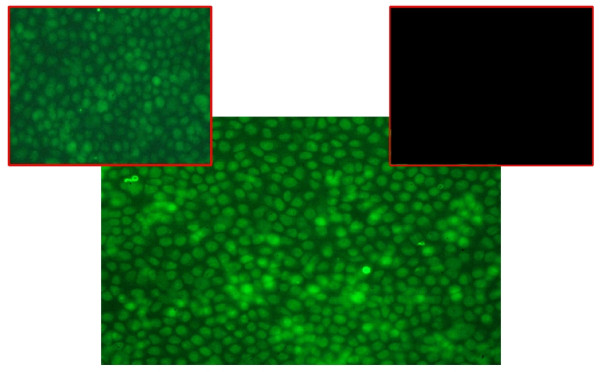
**An extensive DNA damage is caused by treatment with PD166866**. After treatment with the drug (50 μM for 24 hours), the cell nuclei were permeabilized. Fluorescent dUTP and terminal-deoxynucleotide-transferase were added. The enzyme conjugates the nucleotide where the sugar-phosphate backbone is interrupted. The high intensity of fluorescence (large panel) indicated of extensive DNA damage due to the exposure to the drug. This is also monitored in cells treated with H_2_O_2 _(small left panel), while it is virtually absent in untreated control cells (small right panel).

The cells were treated with PD166866 in the usual experimental conditions (50 μM for 24 hours). Results show a very evident fluorescent staining of the cells treated with the drug (Figure 3, large panel) which is a sign of extensive DNA rupturing. In the positive control, cells treated with H_2_O_2 _also a very diffuse fluorescence is visible (Figure 3, left small panel). On the contrary, little if any fluorescence is monitored in control plates (Figure 3, right small panel). Therefore we can conclude that in cells treated for 24 hours with PD166866 the apoptotic pathway is in progress.

### Poly-ADP-Ribose-Polymerase (PARP) is accumulated after treatment with PD166866

The nuclear enzyme PARP-1 catalyzes the transfer and binds ADP-ribose polymers to itself and other nuclear proteins in response to interruptions of the DNA phosphate backbone. The caspases are primarily involved in the cleavage of PARP-1 into two fragments and this has become a general hallmark of apoptosis [25-29].

**Figure 4:**
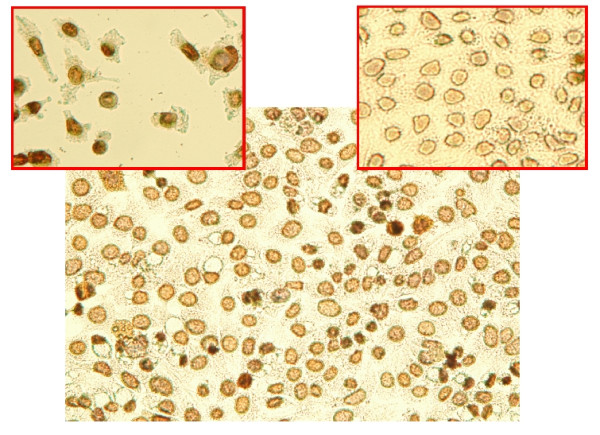
**Accumulation Poly-ADP-Ribose-Polymerase (PARP) in cells treated with PD166866 evidenced by imuno-histochemistry**. Cells were treated with the drug (50 μM for 24 hours) and processed by immuno-histochemical techniques to visualize the intracellular accumulation of PARP. The dark nuclei indicate accumulation of this enzyme in treated cells (large panel). The immuno-reaction also occurs in positive control cells treated with H_2_0_2 _(left small panel) while it is almost absent in untreated control cells. These results indicate cell death.

Results of Figure 4 (large panel) show a relevant immuno-positivity to PARP-1 in cells treated with PD166866 (24 hours 50 μM) which is also monitored in positive control cells where apoptosis was caused by the administration of H_2_O_2 _(upper left panel). The overall conclusion is that the cells treated with the drug are found actually in a condition of advanced apoptosis.

## Discussion

The family of Growth Factor Receptors (FGFR) is constituted by tyrosine kinases involved in a number of different cell functions ranging from cell growth control to mytogenesis and differentiation. Consequently, the interruption of the tyrosine-kinase signal transduction is considered a powerful strategy to inhibit angiogenesis and tumor cell proliferation: therefore fibroblast growth factors and their high-affinity receptors play a crucial role for cell growth survival and maintenance. The interplay between growth factors and their receptors is indeed a very complex one; however, the overall emerging picture is that PD166866, as a tyrosine kinase inhibitor, is able to invalidate the protective action exerted by different agents inducing apoptosis [30,31]. In any case, inhibition of the FGF receptors mediated by small molecules such as SU5402 and PD166866 have been recently shown to reduce growth, survival and motility, as well as clonogenic potential in non small cancer lung cell lines (SSCLC) [32]. The data reported here indicate that one of the cellular targets of the drug may be the membrane of the HeLa cells which is agreement with the membrane localization of the FGFR. The treatment with PD166866 apparently causes a mitochondrial deficit and an oxidative stress, as demonstrated respectively, by the MTT assay and by the increase of the intracellular concentration of malonyl-dihaldeyde. However, rationalizing how the drug could activate these processes is not an easy task. In any case, the impact of PD166866 on the overall cell metabolism [11] cannot be disregarded as an element of serious perturbation of the cell homeostasis. It may be argued that apoptosis could not be the only death process activated by PD166866. Concerning this particular aspect, the role of PARP in executing the cell death program should be discussed in more detail. In our immunoblotting experiments, PARP-1 was revealed by an antibody directed towards N-terminal fragment of the enzyme thus indicating that proteolytic cleavage, mediated by caspases, actually occurs in our experimental model: therefore DNA repair operated by PARP cannot longer occur and the cells exposed to PD166866 proceed into the apoptotic death.

However, it has been shown that in necrotic death, cleavage of PARP-1 is caspase resistant and its proteolysis is partly or totally caused by lysosomal proteases [33]. Also PARP is not proteolytically cleaved by caspases during apoptosis in hepatocytes [34]. A recent literature report demonstrated that cell death may occur in a caspase-independent manner (CICD, Caspase Independent Cell Death) also defined as necroptosis [35]. Finally, a further form of cell death has been described recently which is distinct from apoptosis, necrosis, or autophagy and is termed parthanatos. This is a PARP-1-dependent ubiquitarious form of cell death involved in all tissues of the organism and in pathologies as diverse as Parkinson's disease, stroke, heart attack, diabetes, and ischemia [36].

The overall conclusion drawn from the evidence presented here is that cells treated with PD166866 mainly die by apoptosis; however the possibility that different forms of cell death may occur contemporarily should be also taken into account. In any case, apart from the mode of death, the results discussed in this work corroborate the idea that PD166866 is able to control in a negative fashion the cell proliferation. With respect to this, the most interesting aspect of the work is that PD166866 is able to inhibit the proliferation of cultured human tumor cells.

## Conclusions

The results presented here show that the synthetic molecule PD166866 has significant anti-proliferative effects. These data were obtained by the colorimetric assay of Mosmann and further validated by vital cell count after trypan blue dying. The TUNEL assay allowed a qualitative assessment of DNA damage which could be one of the reasons leading to cell death: however the possibility of this fluorescent staining to discriminate between apoptosis and necrosis has been long discussed. Therefore we ascertained the type of cell death by immunoprecipitation assays of PARP, enzyme an involved in DNA repair whose expression is enhanced during apoptosis. The extensive immunopositivity monitored in the samples treated with PD166866 allows us to conclude that this drug causes cell death possibly via the activation of the apoptotic pathway, even though other forms of cell death cannot be ruled out. In addition, the results of the lipoperoxidation assays, which indicate an oxidative stress at membrane level, suggest that this cell district could be a target for this molecule. However, further studies are needed to assess the antitumor potential of PD166866: in particular future studies *in vivo*, in animal model systems, are required to elucidate this aspect.

## Competing interests

The authors declare that they have no competing interests.

## Authors' contributions

GR is the group leader and this work represents one of the research lines pursued in his laboratory; he directly supervised the experimental work. MC carried out most of the experimental work. GG and MC contributed with stimulating suggestions and encouraging discussions.
